# Summer Hot Snaps and Winter Conditions: Modelling White Syndrome Outbreaks on Great Barrier Reef Corals

**DOI:** 10.1371/journal.pone.0012210

**Published:** 2010-08-17

**Authors:** Scott F. Heron, Bette L. Willis, William J. Skirving, C. Mark Eakin, Cathie A. Page, Ian R. Miller

**Affiliations:** 1 Coral Reef Watch, National Oceanic and Atmospheric Administration, Townsville, Queensland, Australia; 2 Physics Department and Marine Geophysical Laboratory, School of Engineering and Physical Sciences, James Cook University, Townsville, Queensland, Australia; 3 School of Marine and Tropical Biology and ARC Centre of Excellence for Coral Reef Studies, James Cook University, Townsville, Queensland, Australia; 4 Coral Reef Watch, National Oceanic and Atmospheric Administration, Silver Spring, Maryland, United States of America; 5 Long Term Monitoring Program, Australian Institute of Marine Science, Townsville, Queensland, Australia; Northeastern University, United States of America

## Abstract

Coral reefs are under increasing pressure in a changing climate, one such threat being more frequent and destructive outbreaks of coral diseases. Thermal stress from rising temperatures has been implicated as a causal factor in disease outbreaks observed on the Great Barrier Reef, Australia, and elsewhere in the world. Here, we examine seasonal effects of satellite-derived temperature on the abundance of coral diseases known as white syndromes on the Great Barrier Reef, considering both warm stress during summer and deviations from mean temperatures during the preceding winter. We found a high correlation (r^2^ = 0.953) between summer warm thermal anomalies (Hot Snap) and disease abundance during outbreak events. Inclusion of thermal conditions during the preceding winter revealed that a significant reduction in disease outbreaks occurred following especially cold winters (Cold Snap), potentially related to a reduction in pathogen loading. Furthermore, mild winters (i.e., neither excessively cool nor warm) frequently preceded disease outbreaks. In contrast, disease outbreaks did not typically occur following warm winters, potentially because of increased disease resistance of the coral host. Understanding the balance between the effects of warm and cold winters on disease outbreak will be important in a warming climate. Combining the influence of winter and summer thermal effects resulted in an algorithm that yields both a Seasonal Outlook of disease risk at the conclusion of winter and near real-time monitoring of Outbreak Risk during summer. This satellite-derived system can provide coral reef managers with an assessment of risk three-to-six months in advance of the summer season that can then be refined using near-real-time summer observations. This system can enhance the capacity of managers to prepare for and respond to possible disease outbreaks and focus research efforts to increase understanding of environmental impacts on coral disease in this era of rapidly changing climate.

## Introduction

Disease outbreaks have the potential to cause significant damage to coral reefs, not only as a consequence of widespread mortality of framework-building corals but also because of the consequences for many other dependent reef organisms and the resulting likelihood of phase shifts in community structure [e.g., 1,2]. Evidence from a variety of studies suggests that trends of increasing numbers and severity of damaging coral diseases over the past three decades [3] are linked to temperature anomalies. In particular, disease events have been observed to coincide with or follow episodes of coral bleaching in both the Caribbean and Indo-Pacific reef regions [4]–[7], suggesting links with elevated temperature and/or increased susceptibility of coral hosts. Moreover, a seasonal signal in disease abundance has been detected for a number of coral diseases on the Great Barrier Reef, with temperature being one of the most likely driving factors [4], [8], [9]. A recent modelling study also highlights the likelihood that some coral disease outbreaks are linked to extremes of water temperature, possibly by compounding other factors such as high coral cover [10]. An analysis of the effects of climate change on a number of terrestrial and marine pathogens and their hosts suggests that warming can increase pathogen development and survival, while also increasing host susceptibility [11]. For example, it has been shown that the surface mucus layer, which inhibits pathogen growth on healthy corals, shows diminished antibiotic properties during thermal stress, resulting in lowered disease resistance [12]. This change in the surface microbial community may occur quickly at thresholds that are not yet understood and may persist long after thermal stress ends [13]. Understanding the links between temperature anomalies and coral disease has become paramount given the mounting evidence that temperature anomalies are contributing to the increasing frequency and severity of infectious disease outbreaks in corals globally and to the irrevocable decline of coral reef ecosystems, particularly when coupled with increasing coral bleaching episodes.

Satellite monitoring of sea surface temperature (SST) has been used as the basis for several metrics that evaluate the links between thermal stress and coral bleaching [14], [15]. These metrics provide successful nowcasting of coral bleaching events around the world (e.g., http://coralreefwatch.noaa.gov, http://www.cmar.csiro.au/remotesensing/reeftemp/web/ReefTemp_application.htm), demonstrating the direct link that exists between thermal stress and the breakdown of the coral-*Symbiodinium* symbiotic association known as coral bleaching. The near real-time nature of satellite monitoring provides reef managers with vital information that can enable rapid management response. If similar links exist between disease occurrence and temperature metrics, it should be possible to predict disease outbreak risk based on environmental conditions. Modelling studies can provide a mechanism for exploring the nature of such links, thereby enhancing our understanding of factors promoting disease risk.

Previous modelling [10] used SST in combination with long-term records of disease abundance to identify both coral cover and thermal stress as significant drivers of white syndrome abundance on the Great Barrier Reef (GBR), Australia. Disease risk was predicted using the WSSTA (weekly SST anomaly) metric, which counted the number of weeks during the previous one-year period for which the temperature anomaly was at or above +1°C. The anomaly for each week was calculated by subtracting the long-term average temperature for that week from the measured temperature. The study concluded that a significant proportion of surveys with high disease counts occurred in locations that had experienced five or more weeks of anomalously warm temperatures within the prior year in areas of high (greater than 50%) coral cover. Thermal stress was suggested to be necessary, but not sufficient, to predict outbreak events. However, the WSSTA metric only counts the number of warm anomalies, considering neither the magnitude of warm (positive) temperature anomalies nor any effects of negative anomalies, which may also influence the health of the coral host [e.g. 16,17], its symbiotic algae, or the virulence of pathogens.

A causal relationship has been identified between the coral pathogen *Vibrio coralliilyticus* and coral white syndromes (WS) in locations across the Pacific Ocean [18]. Warm anomalies have been linked to increased populations and virulence of pathogens [19] and the corollary, that cold anomalies may reduce survival, density and virulence of pathogens, has also been proposed [11]. Reductions in WS counts from summer surveys to the following winter surveys at Lizard Island in the northern Great Barrier Reef [8] suggested a role for cold thermal anomalies in disease dynamics. In particular, cold temperature anomalies, especially during winter months, may reduce pathogen loads and thereby the risk of disease outbreaks in the following summer.

Here, we built on existing findings [10] to develop SST metrics that incorporated influences of warm summer anomalies, cold winter anomalies and overall winter conditions to elucidate links between the abundance of coral white syndromes and temperature. We sought a method to predict the risk of disease outbreak based on satellite-derived environmental parameters, the vision being to produce an operationally-available tool for managers. Given the complexity of influences that temperature can have on corals and their pathogens, we explored both positive and negative thermal events, their magnitudes and their relevance during different seasons to produce a decision-tree algorithm.

## Methods

### Field surveys of disease

**Figure 1:**
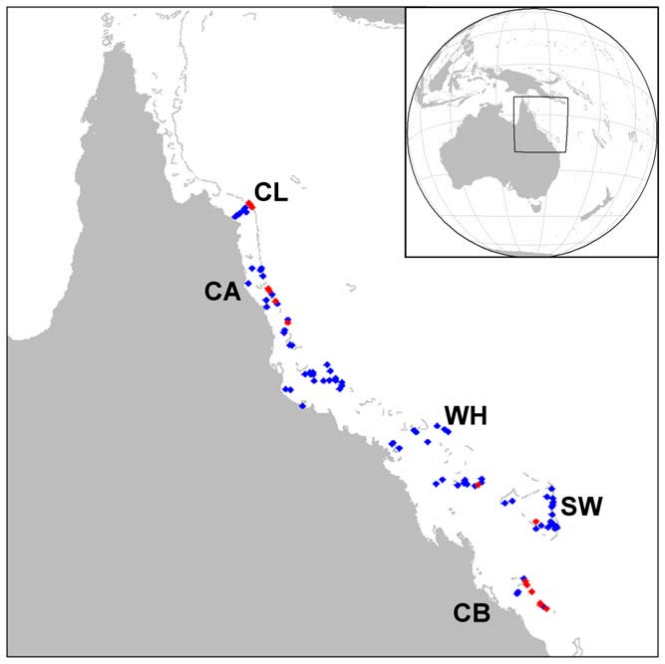
Map of the Great Barrier Reef showing reef locations surveyed for white syndrome abundance. Surveys undertaken as part of the AIMS LTMP and RAP programs. Red symbols indicate locations that experienced a WS outbreak observed during AIMS surveys. Survey sectors indicated for CL = Cooktown-Lizard, CA = Cairns, WH = Whitsunday, SW = Swains and CB = Capricorn-Bunker.

There is a general paucity of long-term disease datasets of the abundance of coral diseases. Due to its longevity and large spatial domain, the Australian Institute of Marine Science's (AIMS) surveys of white syndromes (WS) on the Great Barrier Reef (GBR) [20] provide one of the best datasets with which to explore the links between SST and disease occurrence. For this study, we used field observations of coral disease undertaken by AIMS' Long Term Monitoring Program (LTMP) and Representative Areas Program (RAP) during 1998–2007, under a permit provided by the GBR Marine Park Authority. In total, 47 LTMP and 56 RAP locations were monitored annually or biennially along the length of the GBR, including inner-, mid- and outer-shelf reefs (Fig. 1; see [20] for details of survey timing). Monitoring protocols were identical in these two programs; five belt transects (2 m×50 m) were monitored at three sites for each reef location (total of 1500 m^2^). Transects were permanently marked for repeat visits and photographically sampled along their lengths for post-survey confirmation of data records and a detailed post-analysis of the benthic community, including assessment of benthic structure (percent cover of hard and soft coral to genus, plus other benthic categories such as algae, sponges and substratum type). In some years, transect numbers were reduced at some sites due to weather and/or safety issues.

Data selected for this study were “Total White Syndrome counts” (TWS), a summation of tissue loss observations characterised by a front of recently exposed skeleton coupled with the absence of predators or other visible causative agents, and “percent cover of *Acropora* spp.”, a measure of host density. White syndromes have been reported to be amongst the most prevalent and destructive coral diseases on the GBR. While corals in the TWS category were not identified, acroporid corals are typically the most susceptible to WS [8] and comprise the greatest percentage, by far, of corals in GBR reef assemblages [21], [22]. In cases where the number of transects was reduced from the standard protocol, disease counts were proportionally upscaled to a standard area (counts per 1500 m^2^).

### Defining a disease outbreak

A disease outbreak, or epizootic, has been defined as the occurrence of disease at an unexpected time or place, or at a rate greater than expected [23]. The 10-year WS dataset (1998–2007) spanned years and locations where WS was absent; and years and locations where WS abundance increased up to 20-fold beyond apparent background levels [8]. In the absence of longer-term data from which to establish more rigorous baselines, we defined a threshold for WS outbreaks across all locations by statistically isolating unusually high disease events. Our definition assumed that (a) outbreaks did not occur in all years within the record; and (b) outbreaks did not occur at all survey locations in outbreak years. These assumptions were reasonable given the temporal and geographic extent (>1700 km along the length of the GBR) of the surveys.

To identify the outbreak threshold in the WS dataset, the maximum observed disease abundance was selected for each year and the overall mean and standard deviation of these maxima calculated. Outliers (i.e., outbreaks) were defined as maximum abundance values that were greater than the overall mean value plus one standard deviation. Any such outliers were replaced with the next highest abundance value for that year and the overall mean and standard deviation recalculated. This process was iterated until no outliers existed, with the outbreak threshold defined as the sum of the final values of overall mean and standard deviation of the maxima, with all excluded outliers thus defined as outbreaks.

### Temperature-based parameters

A previous study [10] used the retrospective Pathfinder v5.0 SST dataset [24], at ∼4 km spatial and weekly temporal resolution. The data were temporally gap-filled using a simple interpolation if cloud or other algorithmic tests deemed the quality of a SST value to be poor. Here we also utilised the Pathfinder v5.0 dataset as the source of temperature data but used only night-time retrievals as these are generally more representative of temperature variability at the depths of corals [25]. Additionally, we employed a more sophisticated gap-filling technique than that employed previously [10] for data deemed to be of poor quality (quality value below four [24]). Data gaps were filled using temporal interpolation only for gaps of 3 weeks or less. Beyond this gap-length, it was considered inappropriate to undertake simple interpolation because of the time-scale of ocean processes. Consequently, any remaining gaps were filled by comparing ambient temperatures in the surrounding pixels with the spatial pattern of climatological temperatures (mean for 1985–2005) from the same year-week and setting the gap-value to match the identified pattern. The SST dataset spanned the period 1985–2005 and allowed comparison with the AIMS disease observations for the period 1998–2005.

**Figure 2:**
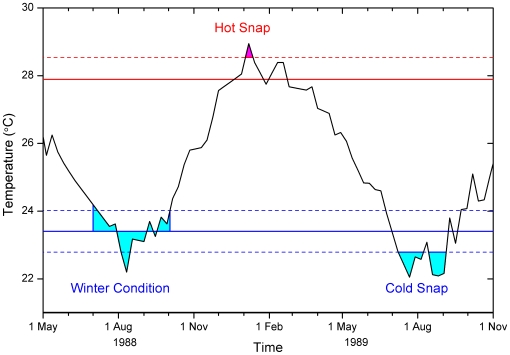
Temperature metrics for a sample temperature time-series. Shown for Slate Reef (149°55′E, 19°40′S). The Hot Snap metric accumulates when temperature exceeds the summer-mean (solid red line) plus one summer-standard-deviation (dashed red line). The Winter Condition metric accumulates anomalies with respect to the winter mean (solid-blue line) that are within the three winter months and/or below the winter-mean plus one winter-standard-deviation (dashed blue line). The Cold Snap metric accumulates when temperature drops below the winter-mean less one winter-standard-deviation (dashed blue line).

Several new metrics of environmental conditions were developed to compare with *in situ* WS abundance data and to improve upon the WSSTA metric [10]. A variety of additional temperature-based metrics were examined (including maximum and minimum temperature; maximum and minimum anomaly; and temperature events above or below various thresholds); the three metrics that provided significant predictive capability are presented here. These metrics incorporated both the magnitude and duration of anomalous thermal events by integrating temperature anomalies through time; thus their units are °C-weeks. For each metric, we calculated anomalies from a temperature *baseline* and summed anomalies through a *period of accumulation* (see example in Fig. 2).

The Hot Snap metric examined whether unusually warm conditions were experienced during the summer period preceding each disease survey. A pixel-by-pixel summer mean temperature was constructed by averaging all SST values from the three climatologically warmest months. The summer standard deviation was also calculated for each pixel and used to identify significantly warm excursions from the summer mean. Hot Snaps occurred when the temperature exceeded the *baseline*, defined for Hot Snaps as one standard deviation above the summer mean. The *period of accumulation* included dates from three months before the most-recent summer that preceded each survey, through to the date of the survey. We accumulated temperatures exceeding the summer baseline during this period, including values outside the climatologically warmest months to incorporate any extra-seasonal warming. As only positive anomalies contribute to the Hot Snap metric, heat stress accumulations at the time of the disease surveys were either positive or zero.

The Cold Snap metric is essentially the winter-time corollary to the Hot Snap, combining the magnitude and duration of cold events prior to the summer accumulation. Pixel-by-pixel winter mean temperatures and standard deviations were determined from all SST values that occurred during the three coldest months. The Cold Snap *baseline* was set at one winter standard deviation below the winter mean. The *period of accumulation* was for the nine-months that preceded the most-recent summer (i.e., Cold Snap is calculated prior to Hot Snap). Temperatures below this baseline, including any anomalously cold temperatures outside the defined winter months, were accumulated. This tested the hypothesis that anomalous cold conditions have a negative impact on pathogens, reducing the risk of disease outbreaks. Only negative anomalies (i.e., temperatures less than the baseline) contribute to the Cold Snap metric, resulting in either negative (anomalously cold) or zero metric values.

The Winter Condition metric provided an alternate measure of winter pre-conditioning, measuring the overall conditions of the cooler months in the annual temperature cycle. This metric was designed to determine if the winter was unusually cold or warm, potentially affecting pathogen loads and/or host susceptibility and thus the risk of disease should subsequent warm summer stress occur. Using the same winter means and standard deviations calculated for the Cold Snap metric, the Winter Condition *baseline* was set to the winter mean temperature. The *period of accumulation* included (a) any time when the temperature was at or below the winter mean plus one standard deviation, to include all winter-like conditions; and (b) the three winter months, even during times when temperatures exceeded one standard deviation above the winter mean – capturing unusually warm periods during winter. Anomalies calculated with respect to the Winter Condition *baseline* could therefore be either negative or positive. All anomalies in this period were accumulated in the Winter Condition metric, giving an overall measure of winter conditions that could be either positive or negative relative to the mean climatology.

As the Pathfinder SST dataset is reprocessed retrospectively; i.e., the data are not produced in real-time, a different dataset would be needed for a near real-time product. NOAA Coral Reef Watch currently produces its near real-time Operational SST twice each week and at ∼50 km (0.5°) spatial resolution for the global ocean [25]. The Operational SST uses the same split-window algorithm as the Pathfinder dataset but a different cloud-clearing methodology. We tested the potential of using the above metrics to provide near real-time risk assessments with NOAA's operational products by evaluating Hot Snap, Cold Snap and Winter Condition metrics calculated using a 50 km, twice-weekly SST dataset. These 50 km data were derived from the same 4 km Pathfinder data as above, but sub-sampled using an algorithm similar to the one used for the near real-time data [26].

### Developing an outbreak risk algorithm

WS outbreaks have been shown to be dependent on multiple environmental factors as well as biotic factors like host density [e.g., 10], reflecting the role that interactions between coral hosts and pathogens play in disease causation [23]. As the SST metrics focused on three different aspects of temperature stress, each was tested independently to evaluate its role in describing disease risk. Algorithms were then developed to amalgamate different combinations of information from these metrics into a decision tree system [27] to predict outbreak risk. WS outbreaks were hindcast using 4 km and 50 km SST metrics to compare the system outcomes at retrospective and near real-time global spatial resolutions, respectively.

## Results

Overlap of disease survey dates with periods of satellite SST data acquisition between 1998 and 2005 provided a dataset of 342 data points with which to explore the relationship between WS abundance and thermal anomalies. January through March were the three warmest (i.e., summer) months at all survey locations. Similarly, the three coldest months were also consecutive (Jul-Sep at all but one survey location), thereby enabling identification of continuous seasons for each location. Based on these empirically-derived definitions of seasonal timing, 84 of the disease surveys occurred in autumn, 104 in winter, 107 in spring and 47 in summer. *Acropora* spp. coral cover across the 342 surveys was 14.0±16.7% (mean ± standard deviation). Based on this variability we set the threshold for high coral cover at 30% (mean plus one standard deviation).

### Defining a disease outbreak

**Figure 3:**
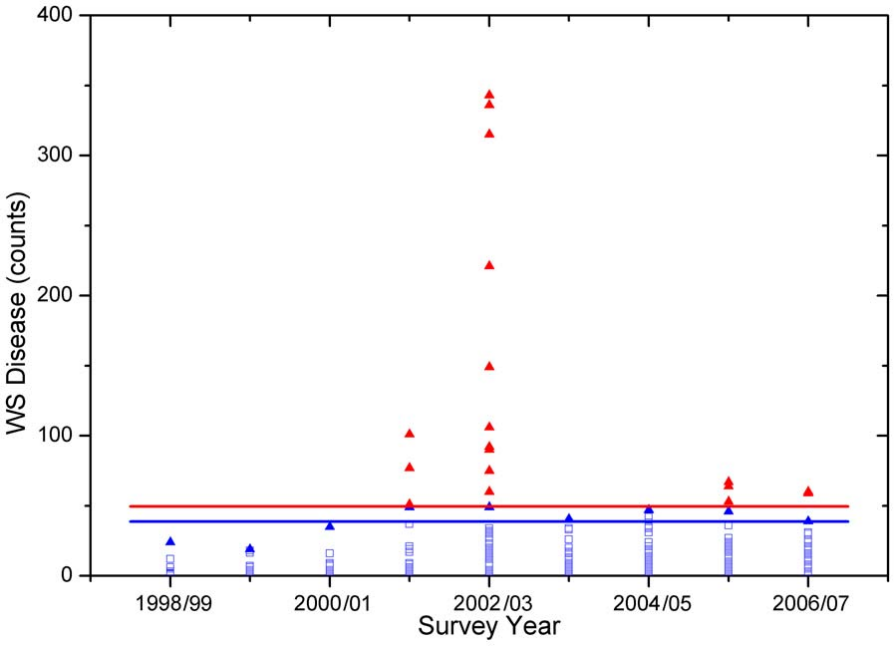
White syndrome abundance plotted against survey years, showing outbreak events (red triangle). Outbreak threshold determined by the iterative analysis (see Methods). Open blue squares correspond to disease observations interpreted as non-outbreak abundances, with the yearly-maximum non-outbreak events, all within one standard deviation of their mean, marked by blue triangles. The blue line shows the mean of non-outbreak yearly maxima (38.7 WS cases per 1500 m^2^); the red line is the outbreak threshold, one standard deviation (10.9 WS cases per 1500 m^2^) above the mean.

**Figure 4:**
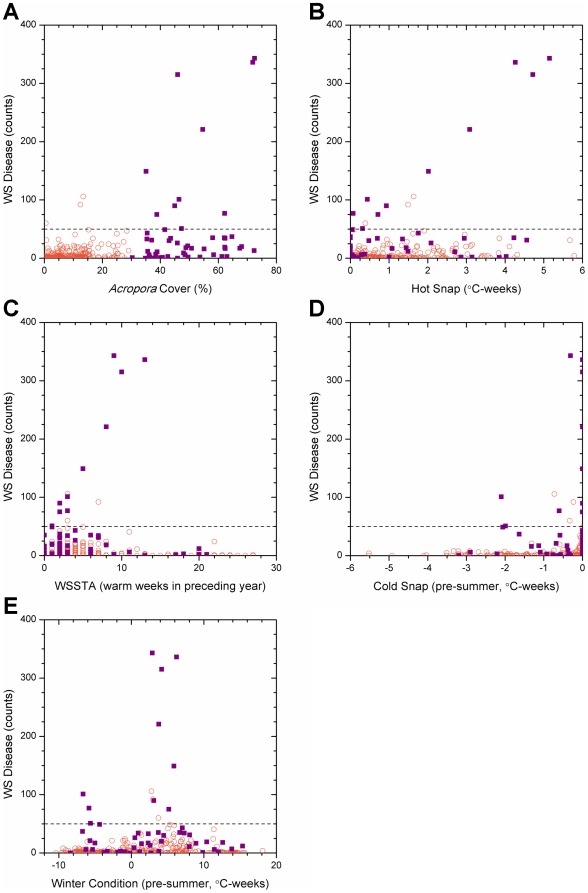
Variation in white syndrome disease counts with coral cover and 4 km satellite metrics. The symbol shape and colour indicate whether *Acropora* spp. coral cover was low: <30% (open orange circle), or high: ≥30% (violet square). Dashed lines indicate the outbreak threshold (50 WS cases per 1500 m^2^). WS counts plotted against (a) *Acropora* spp. cover; (b) Hot Snap; (c) WSSTA; (d) Cold Snap; and (e) Winter Condition.

The mean of annual maximum WS abundance values, excluding outliers (see Methods), was 38.7 WS cases per 1500 m^2^ (standard survey area), with a standard deviation of 10.9 cases per 1500 m^2^ (Fig. 3). Based on the iterative approach we used to calculate this mean and standard deviation, we defined a WS outbreak at these GBR locations to occur when disease counts reached or exceeded 50 cases per 1500 m^2^; i.e., when the number of WS cases was greater than the mean of the annual maxima plus one SD. This value roughly coincided with the upper boundary of a data cloud that stretched across the range of *Acropora* cover and Hot Snap values and was generally separated from high disease abundance values clearly associated with outbreaks (Fig. 4a,b), thus supporting its use as a threshold outbreak value. Based on this threshold, WS outbreaks were recorded in 13 surveys (3.8%) between 1998 and 2005 (the period of comparison with satellite data). All outbreaks occurred in 2001 and 2002 on mid- and outer-shelf reefs in the northern and southern GBR (locations marked in Fig. 1). The great majority of outbreaks were detected during winter month surveys (n = 10 surveys), with the remaining three outbreaks detected during spring surveys. The higher frequency of WS outbreaks detected in winter surveys may reflect the two-fold greater number of surveys completed in winter compared with summer months. *Acropora* coral cover was high (≥30%) at 10 of the 13 outbreak locations.

Scatter plots of disease counts against *Acropora* spp. coral cover (Fig. 4a) and 4 km satellite-derived metrics (Figs. 4b-e) highlight the large number of sites with low or zero abundance of WS recorded in disease surveys. Considering only the surveys in which WS outbreaks were recorded, a positive correlation (r^2^ = 0.36) was detected between percent cover of *Acropora* spp. and WS abundance (Fig. 4a), thus the highest disease counts occurred in regions with highest cover of host corals.

### Temperature-based parameters

The full suite of temperature-based metrics developed in this study was compared with disease abundance; the results of only those metrics found to have predictive skill are described below.

For sites experiencing WS outbreaks (≥50 cases per 1500 m^2^), there was also a strong linear relationship between the Hot Snap metric and WS abundance (WS = 39.0+59.8 * Hot Snap; r^2^ = 0.953; Fig. 4b), suggesting a link between warm temperature stress and WS outbreaks. Note that the intercept value agreed surprisingly well with the mean of annual maximum abundance values, excluding outliers, lending support to the threshold for outbreaks defined in this study. The highest WS abundances occurred concurrently with the highest Hot Snap values recorded, in contrast to the pattern found for the WSSTA metric [10] (Fig. 4c). In the latter case, the highest WS abundances were recorded when the WSSTA metric was approximately half of its maximum value, whereas low or zero WS abundances were recorded when the WSSTA metric peaked, suggesting that the Hot Snap metric provides a better measure of heat stress experienced by corals than WSSTA. While the correlation between WS outbreaks and the Hot Snap metric (r^2^ = 0.953) was marginally higher than that with WSSTA (r^2^ = 0.834), positive values of both metrics successfully predicted the 13 high-disease events. However, surveys revealed a large number of low-disease events when both metrics showed high thermal stress, suggesting that neither was sufficient to predict low-disease counts: of 329 low-disease events, a Hot Snap of 0°C-weeks correctly predicted 92 events, while WSSTA  = 0 predicted only 65. Raising the metric threshold to WSSTA ≥5 [10] correctly identified more low-disease counts (216 of 329) at the cost of successfully predicting high-disease events (6 of 13); a similar pattern was found for Hot Snap ≥1°C-weeks (214 of 329 and 7 of 13, respectively). Thus, observed WS outbreaks were preceded by Hot Snaps but anomalously warm summer conditions were not sufficient for outbreaks to occur.

Analysis of the Cold Snap metric (Fig. 4d) indicated that the highest WS abundances occurred in the absence of unusually cold temperatures during winter. Of the 13 WS outbreaks, nine occurred following winters when cold snaps did not occur (Cold Snap ≥−0.5°C-weeks). The corollary was also true; lower levels of disease occurred following winters characterised by Cold Snaps (<−0.5°C-weeks), suggesting that unusually cold periods hindered WS outbreaks. The remaining WS outbreaks occurred at Cold Snap values around −2.0°C-weeks, with considerably lower WS abundance than the highest disease counts. Thus, WS outbreaks were predominately seen after winters without significant Cold Snaps.

The Winter Condition scatter plot (Fig. 4e) shows that the distribution of disease counts was offset slightly to the right of the mean baseline Winter Condition (warm bias). The highest disease counts coincided with Winter Condition values of 2.5–6.5°C-weeks, values that were at the centre of the observed range of this metric across all surveys [range: −11 to +19]. The majority of WS outbreak observations (10 of 13) occurred within this central peak, suggesting that mild winters (i.e., those that were neither unusually warm nor unusually cool) may have facilitated outbreaks of WS. The corollary to this considered the 329 non-outbreak surveys, of which 273 experienced values outside the central peak of the Winter Condition distribution. This suggested that disease outbreaks were inhibited when preceded by either cooler or warmer winters. The three outbreaks outside the central peak were grouped at Winter Condition values around −6°C-weeks; this grouping was inconsistent with an apparent Gaussian envelope encompassing the rest of the distribution. Thus, WS outbreaks predominately were seen following mild winters, infrequently after unusually cool winters and were not observed to occur following warm winters.

Repeating the calculation of the metrics using the 50 km, twice-weekly SST data yielded similar results (see Fig. S1 in Supporting Online Material). The strong linear relationship between high-disease and Hot Snaps remained (r^2^ = 0.878), as did the association between high-disease events and the absence of Cold Snaps. The peak in the Winter Condition metric was closer to zero, likely a function of the warm-bias sub-sampling algorithm giving consistently increased temperature in the 50 km metrics compared to the 4 km metrics. The mild Winter Condition for the 50 km satellite data was slightly cooler (1.0–5.0°C-weeks) than that found for the 4 km Winter Condition. While variability was slightly higher than for the 4 km metrics, the predictive capacity of the metrics at 50 km resolution remains strong.

### Developing an outbreak risk algorithm

Using our defined threshold for disease outbreak (≥50 WS cases per 1500 m^2^), we increased the effectiveness of predicting outbreak risk by combining the outcomes from our three satellite metrics into a decision tree system. A successful predictive tool should forecast the likelihood of both high- and low-disease abundance. While *Acropora* spp. coral cover cannot be detected through remote sensing, the threshold of ≥30% provided guidance in identifying reefs for which this system could be expected to perform properly. For reefs that met or exceeded 30% coral cover, the following system provided the greatest success at predicting WS outbreaks: (1) a Seasonal Outlook using only winter metrics to predict risk 3–6 months in advance; and (2) a near real-time Outbreak Risk assessment during the summer warm period.

First, the Seasonal Outlook algorithm evaluated the two winter metrics. Locations that experienced non-mild winters (Winter Condition metric outside the range 2.5–6.5°C-weeks) and/or experienced a Cold Snap were assigned as having “No Risk” of disease outbreak. Locations that experienced mild-winters and no Cold Snap (≥−0.5°C-weeks) were assigned to the “At Risk” category for the Seasonal Outlook. Second, those reefs identified as being “At Risk” after the end of the winter were monitored for their summer Outbreak Risk. For locations that experienced mild-winters and no Cold Snap, the near real-time Outbreak Risk was assigned the value of the Hot Snap metric, of which the range was 0–6°C-weeks for low to high risk.

(1) *Seasonal Outlook (3–6 month lead-time)*

 Mild Winter Condition:
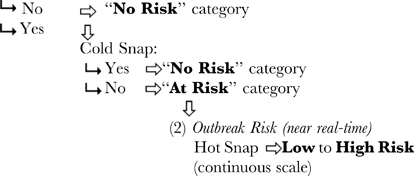



**Figure 5:**
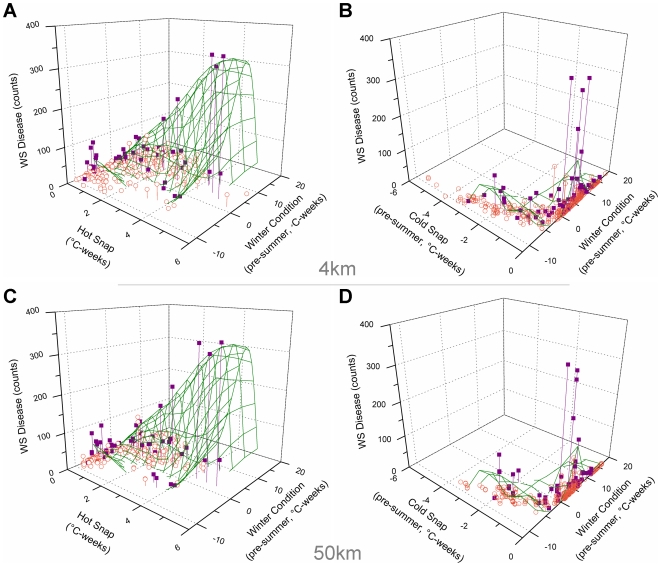
Observed disease counts plotted against satellite metrics. (a) 4 km summer-stress monitoring metrics; (b) 4 km post-winter seasonal forecast metrics; (c) 50 km summer-stress monitoring metrics; and (d) 50 km post-winter seasonal forecast metrics. Coral cover of *Acropora* spp. is indicated by open orange circles (<30%); and violet squares (≥30%).

**Figure 6:**
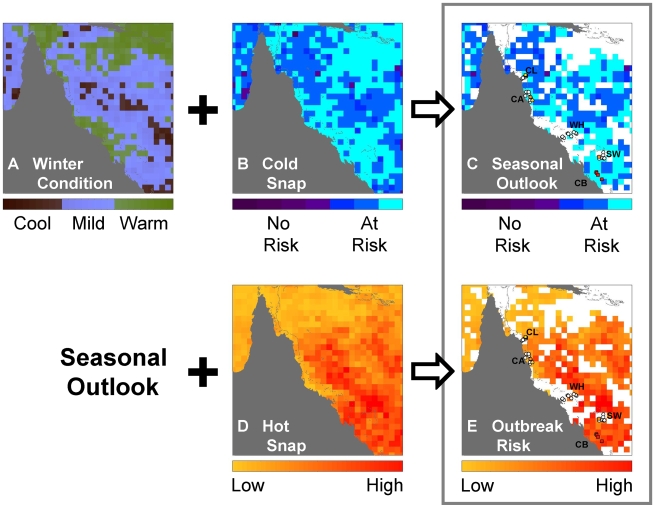
Maps of metrics and risk assessments for 2001-02. (a) Winter Condition for winter 2001; (b) Cold Snap for winter 2001; (c) Seasonal Outlook – Cold Snap spatially filtered by preceding mild-Winter Condition, overlaid with survey data [circle: *Acropora* cover <30%, square: *Acropora* cover ≥30%; white: disease <50 counts, yellow: 50≤ disease <100 counts, orange: 100≤ disease <200 counts, red: disease ≥200 counts]. Survey sectors indicated per Fig. 1; (d) Hot Snap for summer 2001-02; (e) Near real-time Outbreak Risk – Hot Snap spatially filtered by preceding mild-Winter Condition and/or Cold Snap event, overlaid with survey data [as for (c)]. The grey box encloses the final risk assessment products.

## Discussion

Use of modelling to explore the relationship between seasonal temperature anomalies and coral disease abundance has revealed the importance of both warm summer and cold winter temperature anomalies in explaining patterns of white syndrome abundance on the Great Barrier Reef. WS outbreaks showed clear relationships with all three temperature metrics developed; i.e., with stressfully warm summer periods, with a lack of unusually cold conditions during the preceding winter and with overall mild conditions during the preceding winter. However, a combination of the three metrics in a decision tree system yielded the greatest potential for predicting WS outbreak risk.

The significant correlation between Hot Snaps and WS abundance confirmed that WS outbreaks typically occur following anomalously warm summer periods. The Hot Snap metric provided an improvement over the WSSTA metric [10] in describing WS abundance, indicating that a simple count of warm weeks did not adequately characterise outbreak risk. This improvement likely resulted from the inclusion of both magnitude and duration of the anomaly in the Hot Snap metric and from accumulating warm stress only during the summer months, rather than through an entire year. The WSSTA metric included warmer-than-usual periods outside summer, which may have had an inverse influence on disease occurrence. Based on evaluation of the Winter Condition metric, warm periods during winter appear to reduce the likelihood of disease events, possibly through mechanisms such as increased host resistance. Thus, inclusion of only summer warm anomalies in the Hot Snap metric probably enhanced its sensitivity as a predictor of WS. For both Hot Snap and WSSTA metrics, high (>30%) cover of *Acropora* spp. seemed to be a necessary pre-condition for WS outbreaks. However, while both warm temperature and high *Acropora* spp. cover appeared to be necessary for WS outbreaks, these did not completely explain temporal patterns of disease occurrence. In particular, there were several cases of low WS abundance when the metrics were high. Thus additional factors were needed to develop a robust prediction of outbreak risk.

Inclusion of the winter metrics in a conditional manner in the algorithm significantly reduced the number of false outbreak predictions. Most (9 of 13) disease outbreaks in surveys followed winters with few or no cold anomalies (i.e., Cold Snap ≥−0.5°C-weeks). This was consistent with the hypothesis that cold winters reduced WS abundance perhaps by reducing pathogen loads. The data did not support the alternative hypothesis that cold stress could have increased the susceptibility of corals to WS, although further study will be needed to verify this. The correlation between Cold Snaps and low-disease occurrence was an important result for understanding the influence of temperature on disease abundance and for the prediction of disease outbreaks. An exception to this general pattern was seen in a small group of disease observations that corresponded to Cold Snap of *ca*. −2.0°C-weeks. These surveys occurred during winter and were therefore being compared with Cold Snap values from almost one year before the surveys (i.e., before the preceding summer). This large time interval made it likely that other mechanisms intervened to exert greater influence on disease abundance, such as the most recent winter-like temperatures. Although we do not have a clear understanding of factors that might have influenced WS abundance in these surveys, we are constrained by the temporal frequency (annual, at best) of the dataset. However, these outbreaks were smaller in magnitude (<120 counts/1500 m^2^) and did occur following a warm summer (Hot Snap >0°C-weeks) and at sites with very high *Acropora* spp. cover (>45%). Thus WS abundance was more likely to have been influenced by conditions during the preceding summer (6 months prior to surveys) than by those of the preceding winter (12 months prior to surveys).

Use of the Winter Condition metric as a pre-condition also significantly improved the predictions from the algorithm. The clustering of WS outbreaks at the centre of the range of Winter Condition values (4.5°C-weeks; Fig. 4e) indicated that most WS outbreaks followed mild winters. Winter Condition values showed a positive offset, likely because early- or late-season winter-like conditions were included within the *period of accumulation*. These periods were most often warmer than the winter-mean temperature. Mild Winter Condition values (2.5–6.5°C-weeks) may increase the potential for pathogens to persist through the winter, providing a larger population from which an outbreak can develop. Low disease abundance coincided with lower (cooler) Winter Condition values supporting the hypothesis that cold winters reduce the likelihood of disease outbreak.

Interestingly, higher (warmer) values of the Winter Condition metric (>6.5°C-weeks) also corresponded with low WS abundance, suggesting that disease outbreaks did not occur following warm winters. Such conditions may have improved host resilience, potentially through mechanisms such as pathogen inhibition as a consequence of antibiotic production [12], which may be facilitated by warmer winters. Most climate change models indicate that winter temperatures will increase more rapidly than summer temperatures [28]. Although winter warming may allow corals to develop stronger disease resistance, increasing winter temperatures would also reduce the likelihood of Cold Snaps that appear to decrease pathogen loads. Patterns in disease abundance over more years and in a greater range of seasonal conditions are needed to evaluate these alternative hypotheses. The three outbreak values seen near a Winter Condition value of −6°C-weeks were from the same winter surveys discussed in the Cold Snap section. As stated above, the inconsistency of these points from the Winter Condition pattern may have resulted from the recent summer season conditions rather than conditions during the prior winter, *ca*. one year earlier.

The greatest utility of the Winter Condition metric was to improve prediction of non-outbreak events at locations and times that did not experience mild winters. As described above, the Hot Snap metric was used to infer the level of risk during summer monitoring at the mild-winter locations. Each of the documented disease outbreaks at high cover locations did coincide with Hot Snap events; however, use of the Hot Snap alone without consideration of the Winter Condition would have yielded a large proportion (71%) of Hot Snap events not associated with outbreaks (falsely predicting an outbreak). When outbreak prediction required pre-conditioning by mild Winter Condition, the rate of false predictions at high-cover locations dropped to 13%. While the mild-winter requirement failed to predict the winter observations discussed above, it successfully selected the most severe outbreaks. There may be a Hot Snap threshold that overrides the requirement for a preceding mild Winter Condition; should this exist, its identification would improve the prediction system. However, we could not identify such a threshold with the existing dataset. Incorporating mild Winter Condition (2.5–6.5°C-weeks) improved the predictive ability of the near real-time assessment of Outbreak Risk during summer (87% accuracy) over the use of summer metrics alone (29% accuracy; Fig. 5a).

Disease risk associated with pathogen loading and the pre-summer condition of the coral host can be assessed in advance of an oncoming warm season. To this end, there is significant value in considering the combination of Winter Condition and Cold Snap metrics to estimate the risk of outbreak leading into the summer period (i.e., a Seasonal Outlook). This combination of metrics can be calculated several months in advance of any subsequent warm stress and indicates pre-conditioning for outbreaks. The rate of false outbreak predictions at high-cover locations was reduced from 79% to 42% by including only locations with mild Winter Condition (Fig. 5b). While the latter rate of erroneous predictions was reduced, it remained too large for the predictive tool to be definitive. However, by providing a 58% success in predicting outbreaks at high-cover sites, the Seasonal Outlook provides a conservative but useful advance notice of disease risk. Even at this skill level, the algorithm provides substantial utility by alerting reef managers to the potential for outbreaks. Additionally, a Seasonal Outlook can inform research by directing survey efforts to target potentially at-risk areas.

The combination of metrics from the winter and summer monitoring produced a system that provided both a Seasonal Outlook at the end of winter and near real-time monitoring of the Outbreak Risk through summer. The example in Fig. 6 shows the condition that was hindcast for 2001–2002 using 50 km metrics. The decision tree identified locations with mild Winter Condition values (2.5–6.5°C-weeks, blue in Fig. 6a) from winter 2001 and assessed risk if there was no Cold Snap (>−0.5°C-weeks, Fig. 6b) to provide a Seasonal Outlook (Fig. 6c), issued at the end of winter 2001. Leading into the subsequent summer (2001–2002), the decision tree employed the Hot Snap values (Fig. 6d) at the at-risk locations from the Seasonal Outlook to produce a map of Outbreak Risk (Fig. 6e). This image would be updated in near real-time as the summer progresses. Surveys conducted within the six-month period centred at 18 Oct 2002 (overlaid on Fig. 6c,e) showed that WS counts in the Capricorn-Bunkers (24°S) exceeded those around Cooktown-Lizard Island (15°S), the Whitsundays (20°S) and in the Swains (22°S), consistent with the patterns in the risk maps. The surveys near Cairns (17°S) all had less then 30% *Acropora* cover and illustrate the need to interpret the Outbreak Risk using local coral community data.

Reducing spatial resolution of the satellite-SST data to 50 km resolution (Fig. 5cd) did not substantially weaken the relationships established between the temperature metrics and WS outbreaks using 4 km data. Given that these quality-checked 4 km data are only available retrospectively, the comparable predictive capacity at the two spatial resolutions suggests that this approach can be implemented to provide a coral disease outbreak risk assessment in near real-time using existing operational data. Managers and monitoring groups can use these to identify locations of potential outbreaks and undertake management responses. While more details on disease transmission and vectors will be needed to fully develop and evaluate management actions, these could include closures to minimize transport of pathogens among reefs and reducing stressors that increase corals' susceptibility to disease.

The statistics used to define the threshold for WS outbreak provide a starting point for the development of thresholds for other diseases and/or locations, although data on the onset and development of a disease in each location would still be required. Such data are needed to further understand the links between temperature variations and disease abundance for each disease type and each coral reef region. We expect that many combinations of diseases and corals will behave differently, however with more data we hope that some common patterns will emerge. The outcomes of this study also highlight the need for long-term monitoring programs to time their surveys to match known or suspected causal factors (e.g., warm summer stress). GBR surveys for WS would be best timed to occur shortly after the end of summer to immediately follow stressful temperatures.

There is also a need to investigate links between disease outbreak and other factors that may influence disease outbreak. These include strong currents and/or wave activity that increase turbulence; the abundance of disease transmission vectors, including fish, divers and vessels; the distance from terrestrial sources of nutrient input; and the time period required to flush the surrounding waters.

The disease data used in this study are from only one group of diseases (white syndromes) and in a region where there is distinct seasonality in the temperature signal. The algorithm needs to be tested and refined against other surveys of this disease for further validation, in this and other locations, and against other diseases that affect corals and other marine organisms before it should be applied widely. However, the findings here are an important step in identifying relationships that may exist between disease outbreaks and the physical environment. More frequent monitoring of all disease types is required to identify interannual and seasonal variations that may be related to variations in temperature. Fortunately, more organizations are now including coral disease monitoring in their survey protocols. With careful experimental design and regular data collection, approaches like the one described here may be able to broadly provide advance warning of coral disease through the use of satellite SST data.

## Supporting Information

Figure S1 Variation in disease counts with 50 km satellite metrics. The symbol shape and colour indicate whether Acropora spp. coral cover was low: <30% (open orange circle), or high: ≥30% (violet square). Dashed lines indicate the outbreak threshold (50 WS cases per 1500 m^2^). WS counts plotted against (a) Hot Snap; (b) Cold Snap; and (c) Winter Condition.
(1.75 MB TIF)
