# Supplementary figures and images for: Summer Hot Snaps and Winter Conditions: Modelling White Syndrome Outbreaks on Great Barrier Reef Corals
Source: PLoS One. 2010 Aug 17;5(8):e12210. doi: 10.1371/journal.pone.0012210 (PMC2923161; doi:10.1371/journal.pone.0012210)

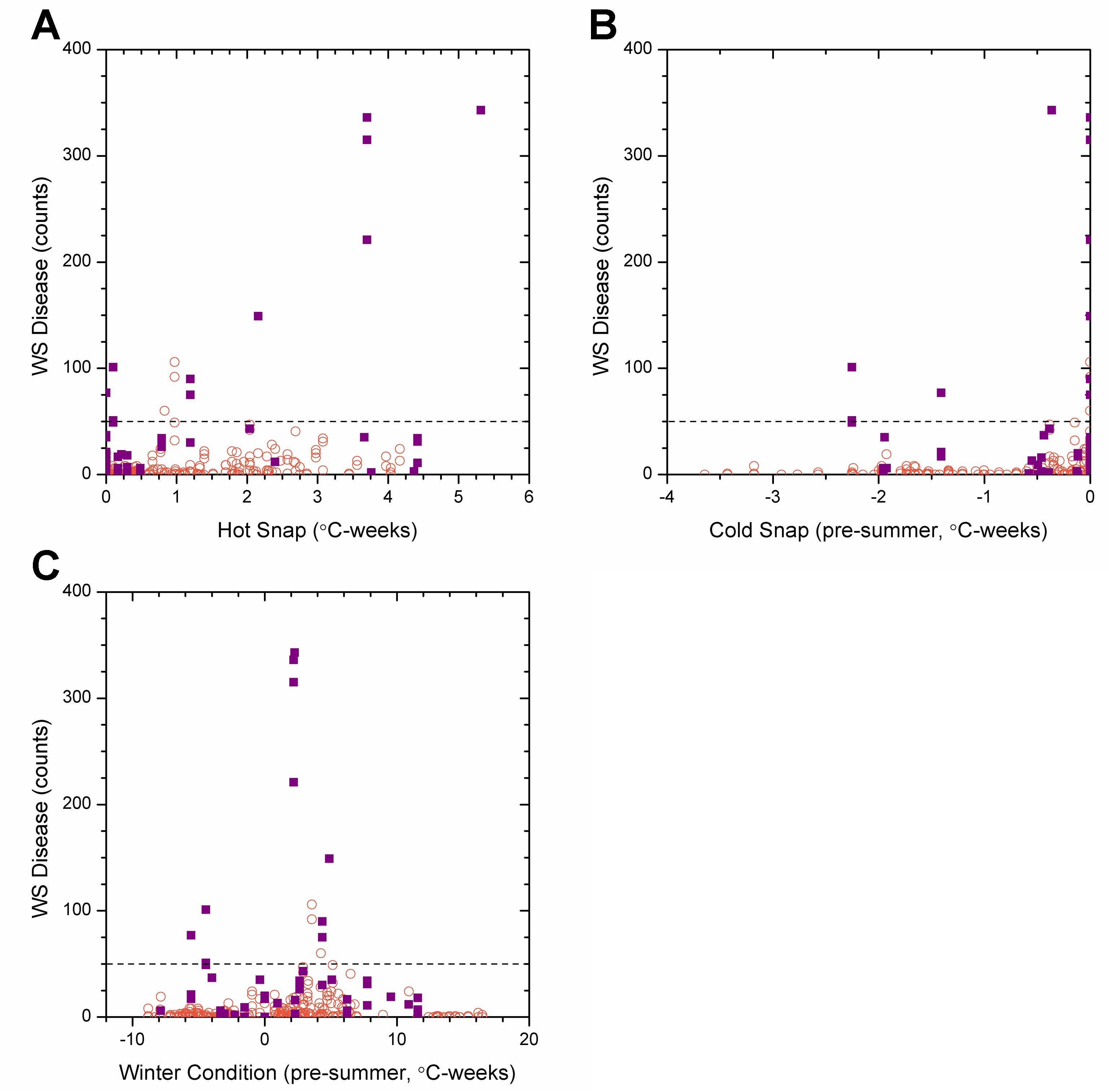

Supplement: Figure S1 — Variation in disease counts with 50 km satellite metrics. The symbol shape and colour indicate whether Acropora spp. coral cover was low: <30% (open orange circle), or high: ≥30% (violet square). Dashed lines indicate the outbreak threshold (50 WS cases per 1500 m2). WS counts plotted against (a) Hot Snap; (b) Cold Snap; and (c) Winter Condition. (1.75 MB TIF) [file pone.0012210.s001.tif]
